# Midsession Reversal Task with Variable Trial Spacings: Further Tests of the Timing Hypothesis with Starlings

**DOI:** 10.1007/s40614-025-00451-7

**Published:** 2025-05-19

**Authors:** Alejandra Salinas, Marco Vasconcelos, Armando Machado

**Affiliations:** 1 William James Center for Research, University of Aveiro, Aveiro, Portugal; 2 Departamento de Educação E Psicologia, Universidade de Aveiro, Campus Universitário de Santiago, 3810-193 Aveiro, Portugal

**Keywords:** Serial reversal learning, Timing, Quantitative analysis, Starlings

## Abstract

This study examined how starlings (*Sturnus unicolor*) adapt to a serial learning task with a predictable reversal in the reinforcement contingencies at midsession. The birds learned a simultaneous discrimination between two options, S1 and S2 (red and green key light colors). Choices of S1 were rewarded during the first 40 trials and choices of S2 were rewarded during the last 40 trials, with variable exponentially distributed ITIs separating the trials. Then, to test the hypothesis that starlings anticipate the midsession reversal based on time into the session, we changed the average of the ITIs during a test session. The hypothesis predicted that with ITIs twice as short during testing, preference would shift from S1 to S2 twice as many trials later than in training, and with ITIs twice as long during testing, preference would shift twice as many trials earlier than in training. Results showed that preference shifted in the predicted direction, but the shifts were smaller in magnitude than predicted. Cumulative difference records plotting choices across time- or trial-into-the-session revealed a variety of adjusting strategies, some consistent with the use of temporal cues, others consistent with the use of local or numerical cues. The variability of strategies occurred both between and within subjects and suggests that multiple cues combine to control behavior in the midsession reversal task.

## Introduction

An animal capable of learning the relation between two successive events can use the first event to prepare for the second. If the second is biologically relevant (food or foe), anticipating its occurrence may have adaptive value. But anticipation may also have costs when the animal, preparing for future contingencies, disregards the current ones. The foregoing issue takes center stage in the Midsession Reversal (MSR) task, a laboratory task used a great deal lately to study serial reversal learning (Cook & Rosen, 2010; McMillan et al., 2017; Rayburn-Reeves et al., 2011, 2016; Stagner et al., 2013; Zentall, 2019, 2020). In a discrete trial session, a hungry subject chooses between two simultaneously available options, S1 and S2. During the first 40 trials, choices of S1, but not of S2, are rewarded with food, whereas during the last 40 trials, choices of S2, but not of S1, are rewarded with food. The reinforcement contingency reverses at midsession.

Researchers have used the MSR task to study how animals adjust their behavior to contingency changes and to identify the cues that guide their actions. They have found that, under some conditions, pigeons and starlings (Machado et al., 2023; Rayburn-Reeves et al., 2011, 2016; Santos et al., 2019, 2021; Soares et al., 2023) and, more rarely, rats (McMillan et al., 2014) anticipate the contingency change. Although the animals choose S1 consistently during the first trials of the session and, for that reason, receive food continuously, they increasingly sample S2 as the reversal trial approaches, indicating anticipation. As a rule, they are indifferent between S1 and S2 close to the reversal trial, and then, after the reversal, preference for S2 continues to grow such that by the end of the session the animals choose S2 consistently and, once again, earn food continuously.

This session-wise response profile is well described by a psychometric function, *p*(n), that relates the probability of choosing S1, *p*, to trial number, n. At first close to 1, *p* decreases before and crosses indifference around the reversal trial. The interpolated value n* for which *p*(n*) = 0.5 defines the Point of Subjective Equality (PSE). After the PSE, *p* continues to decrease to 0 and then remains close to 0 during the last trials. The overall profile resembles a decreasing ogive, wherein anticipation errors correspond to any *p* value less than 1 before the reversal trial, and perseveration errors correspond to any *p* value greater than 0 after the reversal trial. The slope of the psychometric function at the PSE reveals how quickly preference shifts from S1 to S2.

Several studies suggest that pigeons and starlings use a temporal cue to predict the reversal moment (e.g., Laude et al., 2014; Machado et al., 2023; Rayburn-Reeves et al., 2011; 2016; Santos et al., 2019, 2021; Smith et al., 2017; Soares et al., 2023; Zentall, 2020). As trials proceed at a roughly constant pace, the reversal trial occurs at approximately the same time into each session. This temporal regularity allows the animal to learn a temporal differentiation, much as they do in explicit timing tasks such as the peak procedure (Catania, 1970; Roberts, 1981). Some empirical findings support this temporal hypothesis in the MSR task. First, and in general, the observed anticipation errors are consistent with the inaccuracy of the timing process (Weber’s law). Second, and in particular, inserting a temporal gap between two consecutive trials preceding the reversal trial decreases the PSE, and the longer the gap, the greater the decrement (Cook & Rosen, 2010). Third, if the animals learn the MSR task with a fixed ITI and are then tested with a shorter ITI, their PSE in the test session increases with respect to their PSE in the training sessions. On the other hand, if they are tested with a longer ITI, their PSE in the test session decreases. These results are consistent with the temporal hypothesis because an animal that has learned to reverse preference at a particular *time* into the session will reverse preference on a later *trial* if the trials occur at a faster pace, and on an earlier *trial* if the trials occur at a slower pace (McMillan & Roberts, 2012; Smith et al., 2017; Soares et al., 2020, 2023; Zentall, 2020).

The shifts in the PSE observed in studies with pigeons were only partly consistent with the temporal hypothesis. With 10- and 20-min temporal gaps in 50-min sessions the PSE decreased only by four and eight trials, respectively, whereas the temporal hypothesis predicted changes four times as large (Cook & Rosen, 2010). In this case, the direction but not the magnitude of changes in the PSE were consistent with the hypothesis. Likewise, when the ITI was reduced during test sessions, the observed upward shifts in the PSE, although consistent in direction, were much smaller than predicted (Soares et al., 2020). To illustrate, when a session comprises 80 trials, separated by 10-s ITIs, pigeons show a PSE close to 41, the reversal trial (e.g., Soares et al., 2020). If the ITI changes to 5 s during testing (and trials proceed twice as fast), the temporal hypothesis predicts that the PSE will increase by a factor of 2, whereas the empirical findings yielded a much smaller factor of 1.12 to 1.30, with the PSE changing from 40 to about 45 to 55 rather than 80. Finally, when the ITI was doubled during test sessions (and trials proceeded twice as slowly), the observed downward shifts in the PSE were consistent in both direction and magnitude (the PSE decreased by a factor of 2, from about 40 to about 20; Soares et al., 2020).

Machado et al. (2023) argued that some of the discrepancies between hypothesis and data could be due to generalization decrement (Capaldi, 1966), the intrusion of cues during the test session that were absent during training. Consider doubling the trial pace by halving the ITI. An animal relying on timing to reverse its preference would tend to choose S1 until approximately trial 80, only to experience extinction continuously after trial 40. A relatively large number of consecutive extinction trials could cue the animal that the contingencies had changed, and reacting to that novel cue would preempt the timing cue. Instead of shifting preference to S2 around trial 80, the subjects shifted it around trial 50, for example.

The authors also argued that the discrepancy between hypothesis and data does not occur when the ITI doubles because, in this case, generalization decrement is not an issue. When the ITI doubles, the temporal hypothesis predicts a decrease in the PSE from about 40 to about 20, which means that the reinforcement contingencies from trial 1 to trial 20 remain the same in training and testing. No extraneous cue preempts the timing cue.

To test this generalization decrement-based account, two studies changed the reinforcement contingencies during the test session. Machado et al. (2023) trained starlings with the standard MSR task and then, during the test session with ITIs half as long, they (1) rewarded S1 across *all* trials; (2) rewarded S2 after trial 40; and (3) increased the number of trials to 120 to be able to observe the predicted PSE on trial 80. With these conditions, the PSE increased from training to testing by a factor of 1.75 to 2.06. Soares et al. (2023) conducted a similar study with pigeons and found an increase in the PSE by a factor of 1.89. In both studies, the increase was higher than in previous studies and closer to the value predicted by the timing hypothesis.

It is surprising that when the researchers tested with twice as long ITIs and predicted a decrease in the PSE by a factor of 2 (from about 40 to about 20), they observed changes in the PSE by a factor of 1.4 to 1.6 in starlings and of 1.6 in pigeons. In both cases, the decrease was less than in previous studies. Moreover, in both studies, a few birds showed results inconsistent with the temporal hypothesis: In some tests, the PSE did not change. The results of these birds seemed more consistent with a win stay/lose shift strategy or with a number-based anticipation of the reversal trial.

The present study attempted to improve the test of the temporal hypothesis by further reducing the differences between training and testing. One of these differences is the very ITI manipulated to change the trial pace. On the one hand, we need to change the ITI to test the temporal hypothesis, but, on the other hand, we do not want to introduce an easy-to-detect change that reduces the putative influence of the time-into-the-session cue. One way to achieve both goals is to use variable ITIs both in training and testing. To see this, suppose that in an 80-trial MSR task, the ITIs vary randomly according to an exponential distribution with, say, mean 20 s, but such that the reversal trial still occurs at roughly the same time in each session. Assuming animals can learn this modified MSR task, we predict a psychometric function like the one obtained in the standard task, an ogive with a PSE close to the reversal trial and with anticipation and perseveration errors surrounding it. Then, if during the test session, the ITIs also vary randomly but with a mean of 10 s, the difference between training and testing will be harder to detect because the ITI distributions will overlap. Some ITIs from the test session will be shorter and some will be longer than some of the ITIs from training. In the standard MSR task with constant ITIs, no overlap takes place—the change in the ITI is easy to detect from the onset of the test session and its detection may reduce the influence of time-into-the-session on preference reversal. The experiment reported below tested this hypothesis.

We trained starlings in a task with the standard MSR reinforcement contingencies (reward S1 for the first 40 trials and S2 for the last 40 trials), but with variable ITIs averaging either T s or 2 T s. Then, we tested the starlings using variable ITIs averaging either 2 T s or T s, respectively, as well as with the reinforcement contingencies mentioned above to prevent the intrusion of the extinction-based extraneous cues. These two changes—variable ITIs during training and testing, and different reinforcement contingencies during testing—should reduce generalization decrement more than in previous studies and thereby provide a better test of the temporal hypothesis. Also, by extending the MSR task to starlings, we enhance our understanding of the learning processes of a passerine bird used extensively in biological research (Asher & Bateson, 2008), and, in particular, of the cues a high metabolism bird uses to predict changes in reinforcement contingencies.

## Method

### Subjects

Sixteen wild Spotless Starlings (*Sturnus unicolor*) participated in the experiment. The birds were caught in November 2023 near the university campus (capture and holding licenses 504/2023/CAPT and 07/2023/DET, respectively, issued by the Portuguese Department of Wildlife and Conservation). Before the experiment began, the birds were kept together and given unlimited access to food and water. They were housed in pairs in cages that were visually but not acoustically isolated. Twelve cages, divided into six racks, with two cages stacked in each rack, were placed in an experimental room with controlled humidity, temperature (≈20 ºC), and light (12 h:12 h light:dark cycle, with the lights on at 6:00 am and with gradual transitions at dawn and dusk).

Each bird was fitted with a colored plastic leg band for identification purposes and its body weight was recorded under free-feeding conditions (range: 70–100 g). Each bird's weight was subsequently reduced gradually to 90% of its free-feeding weight. Each bird was weighed twice a week. From 6 am to 2 pm, the birds had access to food pellets only in the experimental sessions and as response-contingent rewards (see Apparatus and Procedure below). From 2 to 5 pm, they had free access to five larvae, Orlux (Versele-Laga), chicken feed (Versele-Laga), social interaction with their respective cage partner, and water for drinking and bathing.

By sliding two stainless steel panels through the front wall, each cage could be divided into three compartments, left, center, and right. The left and right compartments were used for experiments, the center compartment was not used. When placing the panels, the experimenter ensured that each starling, identified by its leg band, remained always in the same compartment. The panels were inserted after the free-feeding period, at 5 pm, confining each subject to its compartment and depriving it of food until the first session of the next day. Two bottles of drinking water were always available in each compartment. The starlings remained apart until the last session finished at 2 pm the following day, at which time the panels were removed, and the free-feeding period began.

The experimental protocol was approved by the University's Animal Welfare Committee (B003/2020) and by the Portuguese General Directorate of Food and Veterinary Medicine (0421/000/000/2021). All subjects were released into the wild after participating in two further unrelated experiments.

### Apparatus

Each rack was equipped with two cages mounted vertically, each measuring 135 cm in length, 80 cm in height, and 78.4 cm in depth. Each cage contained a straight rubber mat covering the floor and five perches—three suspended and two on the ground. At the left and right ends of the long cage was the intelligence panel, which comprised three response keys: left, center, and right, each 3.5 cm in length by 3.5 cm in height, positioned 10.7 cm above the floor. The left and right keys were angled at 110° relative to the central key (i.e., "/- \"). Each key could be lit from behind using a 4 × 4 array of LEDs capable of emitting different colors of light. The current experiment used only white, red, and green lights.

Each intelligence panel had a Campden Instruments® pellet dispenser at the back. Upon activation for 300 ms, it delivered a single 20 mg Bio-serv precision pellet into a 4.3 × 3.7 cm opening in the center subpanel, positioned 2.7 cm above the floor. Each pellet release was accompanied by a light flash and an audible sound from the dispenser's motor. All experimental events were coded using Visual Basic running on a Microsoft Windows system. Communications with the experimental chambers were handled through the Whisker interface with 1-ms resolution (Cardinal & Aitken, 2010).

### Procedure

Twelve of the 16 starlings experienced three sessions per day, whereas starlings B3 and B8 completed only two. Although they initially started with three sessions per day, they consistently left one incomplete, so their sessions were reduced to two. Two starlings from Group 1 were subsequently removed from the study because one became sick and the other failed to meet the stability criterion. Each session consisted of a series of trials, and each trial was as follows: The center key blinked with a white light once per second (300 ms on, 700 ms off) and continued to blink until a peck on the key extinguished the light and lit the two side keys, one with a red light and the other with a green light. The colors defined the choice alternatives, S1 and S2. The left–right location of S1 and S2 varied randomly across trials, with the constraint that S1 appeared on the left key on half of the trials. A peck on one of the two alternatives turned both key lights off and initiated the ITI. Moreover, if the choice was correct, a reward of 1 pellet was also delivered.

The experiment used two sets of variable ITIs, exponentially distributed, one with a short average (short ITI) and one with a twice as long average (long ITI). They were constructed using Fleshler and Hoffman’s (1962) algorithm. The algorithm requires two input parameters, the number of different intervals and the average of the intervals, and it returns the list of intervals. For the experiment, we requested 40 different intervals with mean 10 s. Because some of these intervals were very short (< 1 s), we added 3 s to all output intervals. The final list comprised 40 intervals ranging from 3.13 s to 49.89 s and averaging 13 s. This list defined the Short ITIs. To construct the Long ITIs, each value in the Short ITI list was simply doubled. Hence, the Long ITIs ranged from 6.26 s to 99.78 s and averaged 26 s. As a measure of the overlap of the two distributions, we note that, if we sample one interval from each list randomly, the interval from the long ITI list will be smaller than the interval from the short ITI list on about 25% of the trials.

Table 1 shows the experimental design. The starlings were randomly divided into two groups, one with six subjects and the other with eight. The groups differed only in the order they experienced the ITIs. Group 1 learned the task with the Short ITI, whereas Group 2 learned the task with the Long ITI. After the number of training sessions displayed in the table, the birds were always tested with two sessions with the “opposite” ITIs, Long (ITI doubled) if training was with Short, and Short (ITI halved) if training was with Long. We refer to these test sessions, not displayed in Table 1, as Session 1 and Session 2. Each replication of the experiment consisted of two ITI training conditions: Short ITI Condition and Long ITI Condition, and the experiment involved three replications according to an (A-B)-(A-B)-(A-B) design.

**Table 1:** Experimental Design

			Replication 1		Replication 2		Replication 3	
Bird	Group	S1-S2	Short	Long	Short	Long	Short	Long
B1	1	R-G	34	37	19	19	19	34
B2	1	R-G	40	34	19	22	19	19
B16	1	R-G	53	25	19	19	19	25
B6	1	G-R	58	19	19	31	25	28
B12	1	G-R	46	19	19	19	22	19
B15	1	G-R	55	31	19	19	19	31
			Long	Short	Long	Short	Long	Short
B3	2	R-G	86	18	20	24	20	20
B4	2	R-G	55	19	19	19	19	19
B13	2	R-G	61	19	22	19	19	19
B22	2	R-G	46	19	19	28	19	28
B5	2	G-R	50	19	22	19	19	19
B8	2	G-R	42	24	20	28	22	32
B11	2	G-R	43	22	19	19	19	19
B14	2	G-R	55	19	19	31	45	20

For each bird, the table displays its Group, the key light colors assigned to S1 and S2 (R = Red, G = Green), and the number of training sessions with each ITI average (Short = 13 s, Long = 26 s) in each of three Replications. At the end of each training phase there were 2 test sessions with the other ITI average (Doubled ITI after training with Short, and Halved ITI after training with Long)

#### Training Sessions

All training sessions comprised 80 trials with the standard MSR contingencies, S1 choices rewarded during trials 1 to 40, and S2 choices rewarded during trials 41 to 80. The ITIs were obtained by shuffling the (Short or Long) ITI list twice, with the first shuffle yielding the first 40 ITIs and the second shuffle yielding the last 40 ITIs. Because the sum of the 40 ITI values was constant, the reversal trial tended to happen at roughly the same time in each session (i.e., ≈8.7 min with the Short ITIs and ≈17.3 min with the Long ITIs). Sessions ended when the bird completed the 80 trials or when 60 min elapsed. Twelve of the 14 starlings ran three sessions a day. The first session started at 6:30 am, whereas the second and third sessions began 105 min after the previous session ended. Two starlings ran only two sessions a day, also separated by 105 min. Training continued until the bird met the stability criterion of earning at least 80% of the available rewards for three consecutive sessions.

#### Test Sessions

After training with Short ITIs, the birds were tested in two sessions with the same reinforcement contingencies as in training (i.e., S1 rewarded on trials 1 to 40, and S2 rewarded on trials 41 to 80), but with Long ITIs substituting for Short ITIs, which means the ITI was doubled. However, after training with Long ITIs, the test sessions comprised 120 trials. As in training, on trials 1 to 40, only S1 choices were rewarded, but in contrast with training, on trials 41 to 120 both S1 and S2 choices were rewarded. Finally, Short ITIs substituted for Long ITIs, which means the ITI was halved. For each session, the list of Short ITIs was shuffled three times, and the first, second, and third shuffles yielded the ITIs used during the first, second, and third sets of 40 trials. A test session ended when the bird completed the scheduled number of trials (80 or 120) or when the maximum allowed session duration elapsed (60 min for 80-trial test sessions, 90 min for 120-trial test sessions).

For 12 of the 14 starlings, the two test sessions were always the second and third sessions of the day, with the first session being a regular training session. For starlings B3 and B8, the two test sessions replaced the two training sessions. Sessions were conducted seven days a week, and the entire experiment lasted an average of 62 days (range: 51–94).

### Data Analysis

To control circadian effects, the daily sessions were treated as separate blocks and all comparisons were restricted to sessions within the same block, that is, to sessions that started roughly at the same time each day. Results from test Sessions 1 and 2 were compared with the results from the last five training sessions of the same block.

#### Psychometric Function Analysis

For each session, we computed the number of S1 choices in sets of 10 trials. Then, we averaged the functions from the last five training sessions for each bird and averaged the individual functions to obtain group training functions. We expected the usual ogival shape, with *p* close to 1 on the first trials and close to 0 on the last trials, with anticipation and perseveration errors around the reversal trial. We performed a similar analysis on the test sessions and obtained group testing functions. If the temporal hypothesis is correct, the testing function should be to the left of the training function when the testing used longer ITI (Doubled-ITI), and to the right when it used a shorter ITI (Halved-ITI).

To quantify the shifts of the psychometric functions, we computed the PSE for each individual average function using linear interpolation. Then, we calculated the ratio between the PSE of the test session and the average of the PSEs from the last five training sessions of the same block. This ratio defined the PSE Ratio. Finally, we averaged the individual PSE Ratios to obtain the group PSE Ratio and constructed a 95% Confidence Interval (CI) for that average using the *t* distribution. The temporal hypothesis predicts a PSE Ratio close to 0.5 when the ITI is doubled from training to testing, and a PSE Ratio close to 2 when the ITI is halved.

#### Cumulative Difference Record Analysis

The psychometric function and the PSE Ratio inform about performance across trials, not time. But to test the temporal hypothesis directly and somewhat stringently, we need to look at performance both across time and trials. To that end, we used cumulative difference records (Machado et al., 2023). For each session, we computed the difference between the cumulative number of responses to S1 and the cumulative number of responses to S2 as a function of time into the session and as a function of trial number. Let Σ(S1–S2) represent that *cumulative difference*. When Σ(S1–S2) is plotted against time, each new trial moves the record horizontally to the right by the amount of time taken to complete the trial (ITI value + response latencies) and vertically by + 1 or −1 according to whether the bird chose S1 or S2, respectively, on that trial. A positive slope denotes a preference for S1, a negative slope a preference for S2.

**Fig. 1:**
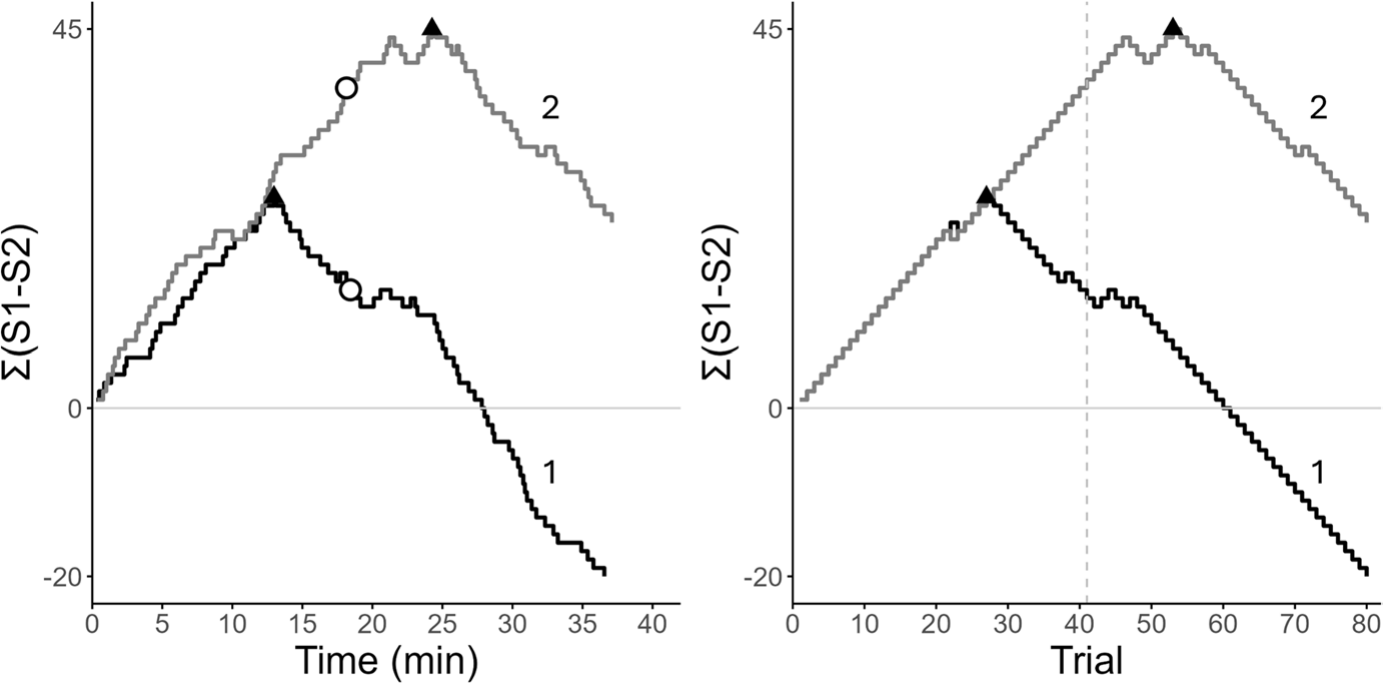
Cumulative Difference Records Plotting the Difference between the Total Number of S1 and S2 choices, Σ(S1-S2), against Time (Left) or Trials (Right). *Note:* Each curve corresponds to a different session. Curves with the same number represent the same session. The filled triangles show the peak of preference, and the empty circles show the contingency reversal moment. The vertical dashed line (right) shows the reversal trial

The left panel of Fig. 1 shows two time-based cumulative difference records. In both, the subject initially prefers S1, and the curves rise with similar slopes. The irregularities are due to variability in the ITIs, as well as to the latencies to start a new trial and to choose a comparison. In curve 1, preference for S1 reaches its maximum at T_max_ ≈13 min (filled triangle) and then falls until the contingency reverses at ≈19 min (empty circle, corresponding to trial 41). A short horizontal segment follows (indifference between S1 and S2), and then the curve falls steadily with a steeper slope. The final negative value of Σ(S1–S2), approximately −20, reveals an overall bias for S2. Curve 2 is similar except that preference reverses after the contingency (T_max_ ≈24 min vs. ≈19 min) and the overall bias is for S1 (final value of Σ(S1–S2) is positive). In these curves, anticipation occurs when preference reverses before the contingency (triangle to the left of the circle), and perseveration occurs when preference reverses after the contingency (triangle to the right of the circle). Optimal performance would show a “∧”-shaped curve, with preference reversing when the contingency reverses (triangle and circle overlap).

The right panel of Fig. 1 shows the same cumulative difference records plotted against trial number; they are trial-based cumulative difference records. Both records show an initial steady preference for S1. For curve 1, the peak of preference occurred on trial N_max_ = 27, before the contingency reversal on trial 41, whereas in curve 2 preference for S1 peaked on trial N_max_ = 53, after the contingency reversal.

To quantify these changes, we performed for T_max_ and N_max_ the same analysis we performed for the PSE: For each session, we computed T_max_ and N_max_, the time and trial at which the time- and trial-based records, respectively, reached their maximum. On ~ 36% of the sessions, the record had the maximum at multiple time points or trials, so we averaged their values. Next, we averaged T_max_ and N_max_ from the last five training sessions and then compared the two averages with T_max_ and N_max_ for the corresponding test session. We refer to the individual ratios as T Ratio and N Ratio. The individual T and N Ratios were then averaged across individuals to obtain group average ratios and to construct 95% Confidence Intervals for the average.

The T and N Ratios allowed us to evaluate whether the observed shifts in preference aligned with the temporal hypothesis. According to the hypothesis, the T Ratios should remain close to 1 whereas the N Ratios (like the PSE Ratios) should yield values close to 0.5 when testing with doubled ITIs, and values close to 2 when testing with halved ITIs. Moreover, if the two indexes, N Ratio, obtained from cumulative difference records, and PSE Ratio, obtained from psychometric functions, provide equally valid indexes of preference reversal, they should be positively correlated. A strong correlation would validate the new N Ratio measure.

To compare the PSE, T and N Ratios, we used mixed ANOVAs with Group as a between-subjects factor (2 levels) and training ITI (2 levels), test Session (2 levels), and Replication (3 levels) as within-subjects factors. We set the significance level at α = 0.01 and use partial-eta square (${\eta }_{p}^{2}$) as a measure of effect size.

## Results

### Psychometric Functions

**Fig. 2:**
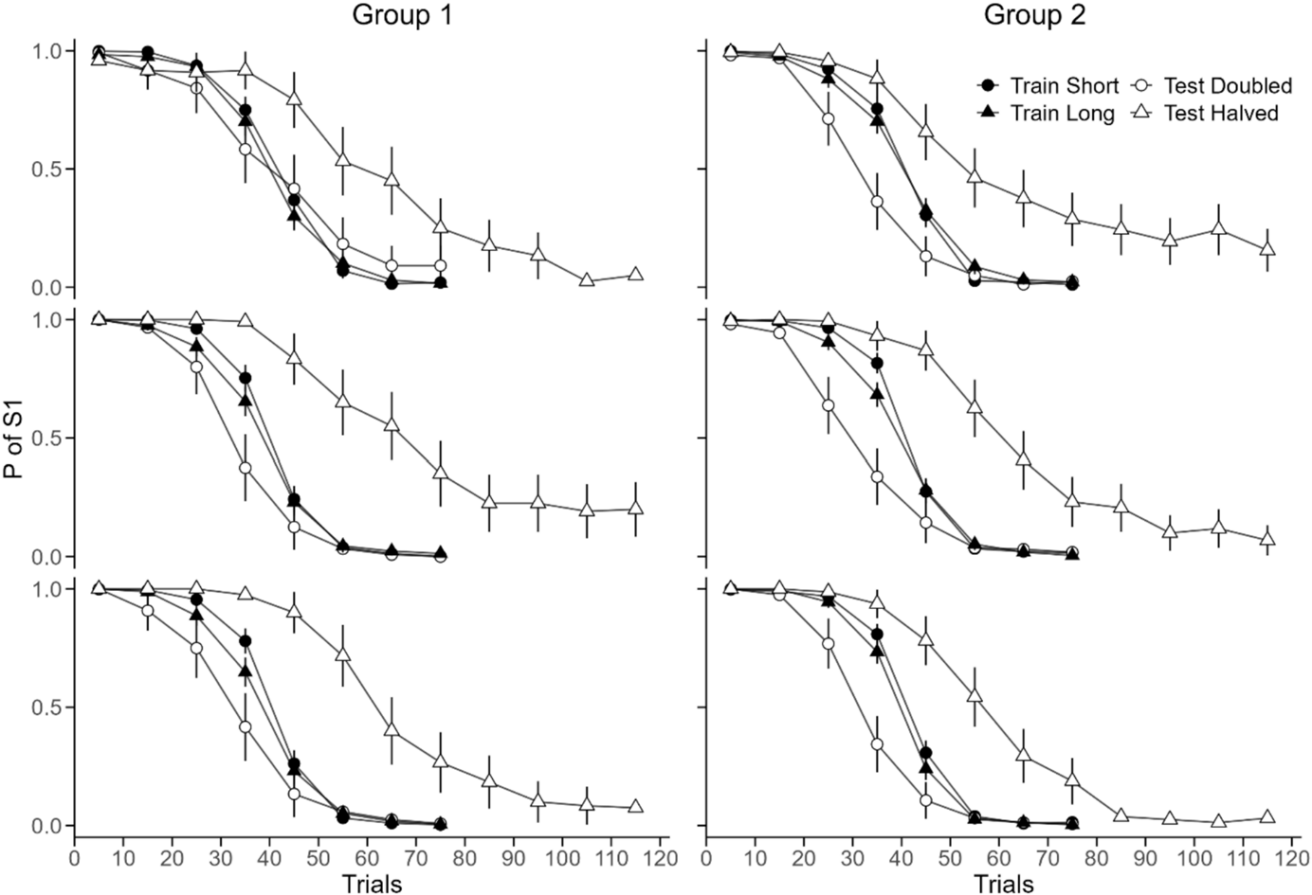
Average Psychometric Functions for Group 1 (Left) and Group 2 (Right) in the Three Replications (Top = Replication 1). *Note.* In each panel, the filled symbols represent the last five training sessions, and the empty symbols represent the two corresponding test sessions. Circles correspond to training with Short and testing with ITI doubled, and triangles correspond to training with Long and testing with ITI halved. Test sessions with ITI halved comprised 120 trials; all other sessions comprised 80 trials. The bars show the SEM

All starlings learned the MSR task, choosing S1 during the first trials and S2 during the last trials. Figure 2 shows the average psychometric functions. The functions had the typical ogival shape both in training and testing and across replications. The overlap of the training curves shows that the birds learned the contingency equally well with variable Short and variable Long ITIs. As with constant ITIs, the curves show anticipation and perseveration errors and indifference close to the contingency reversal trial.

With halved ITIs during testing, we expected the psychometric functions to shift to the right (empty-triangle curve to the right of filled-triangle curve). The shift was observed for both groups and in all replications. The point of indifference during testing increased to 55–65, slightly earlier than predicted by the timing hypothesis (≈80). The second and third replications (middle and bottom panels) showed greater shifts than the initial replication (top), particularly for Group 1.

With doubled ITIs during testing, we expected the psychometric functions to shift to the left (empty-circle curve to the left of filled-circle curve). The shift was observed in all cases except the first replication of Group 1. The indifference points occurred between trials 25 and 30, later than the timing hypothesis predicts (≈20).

**Fig. 3:**
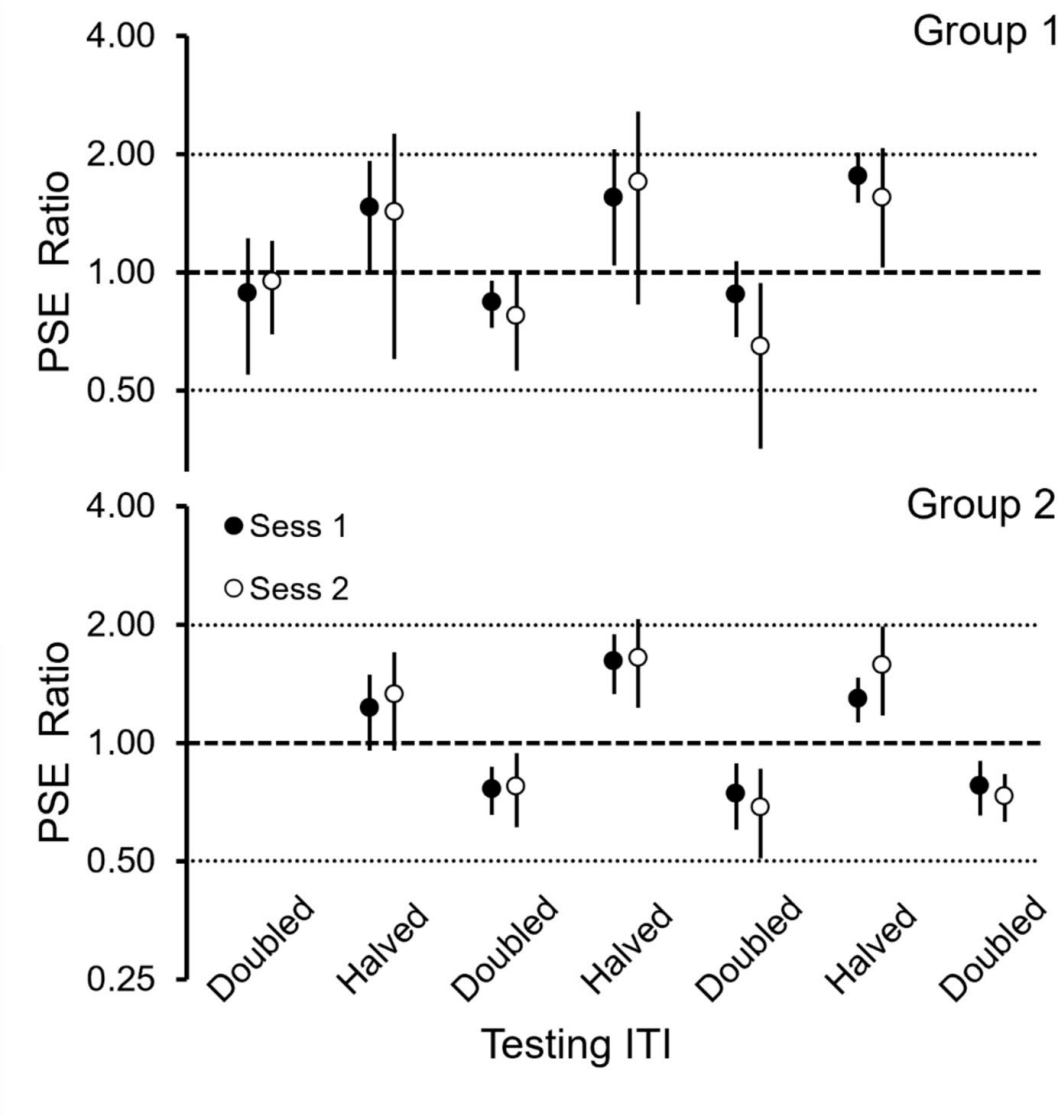
PSE Ratios across the Experiment for Groups 1 (Top) and 2 (Bottom). *Note.* For each group, the six pairs of filled and empty circles show the average PSE Ratio (and 95% CI) for the two test sessions that followed each experimental condition, with condition defined by the ITI used in testing (see X-axis). Doubled and Halved ITIs averaged 26 s and 13 s, respectively. Note logarithmic Y axis

Figure 3 shows the average PSE Ratios obtained in the two test sessions that followed each ITI training condition and in the order the groups experienced them. The dashed line represents no shift in preference from training to testing (ratio ≈1); the two dotted lines represent the predictions of the temporal hypothesis, a ratio ≈0.5 (reversal twice as early) when the ITI was doubled, and a ratio≈2 (reversal twice as late) when the ITI was halved. In 3 of the 168 cases (= 14 birds × 12 conditions per bird), the PSE could not be determined.

For both groups, the average PSE Ratios were consistently greater than 1 when the ITI was halved, and less than 1 when the ITI was doubled. Also, the deviations from 1 were larger in the last two replications than in the first replication. The mixed ANOVA of the PSE Ratios revealed a main effect of the ITI (*F*(1,12) = 47.42, *p* < 0.001) with a strong effect size (${\eta }_{p}^{2}=$ 0.83). In addition, the Replication x ITI interaction also was significant (*F*(2,20) = 6.20, *p* = 0.008), with a moderate effect size (${\eta }_{p}^{2}=$ 0.38). No other effect was significant.

The deviations of the PSE Ratios from 1 were not of the same magnitude. When the testing with ITI was halved the PSE Ratio averaged 1.5, which represents a change of 50% relative to the change predicted by the temporal hypothesis, whereas when the testing with ITI doubled, the PSE Ratio averaged 0.78, which represents a change of 43.7% relative to the change predicted by the temporal hypothesis (i.e., 100(0.78–1)/(0.5–1)).

The 95% CIs had substantial variability, particularly in the smaller Group 1. For this group, of the six CIs in the test with ITI halved, five included 2, the value predicted by the temporal hypothesis; in the Short ITI condition, only one included the predicted value 0.5. For the larger Group 2, the CIs were narrower, and in all but one case they did not include the value predicted by the hypothesis (the exception was in the second ITI halved test, second test session).

### Cumulative Difference Analysis

From each session's cumulative records, we obtained two indices—Tmax and Nmax—representing the time and trial, respectively, at which the preference for S1 peaked. These were calculated for the five sessions preceding the test and the test sessions themselves. During training with short ITIs, T_max_ averaged 9.9 (± 1.3) min, whereas with long ITIs it averaged almost twice as much, 17.8 (± 1.7) min. N_max_, however, remained approximately constant, equal to 40.76 (± 2.45) with Short ITIs, and 38.90 (± 3.76) with Long ITIs, both values slightly shorter than 41, the reversal trial. Taken together, these averages show that starlings learned the MSR task with significantly different trial spacings and session durations.

**Fig. 4:**
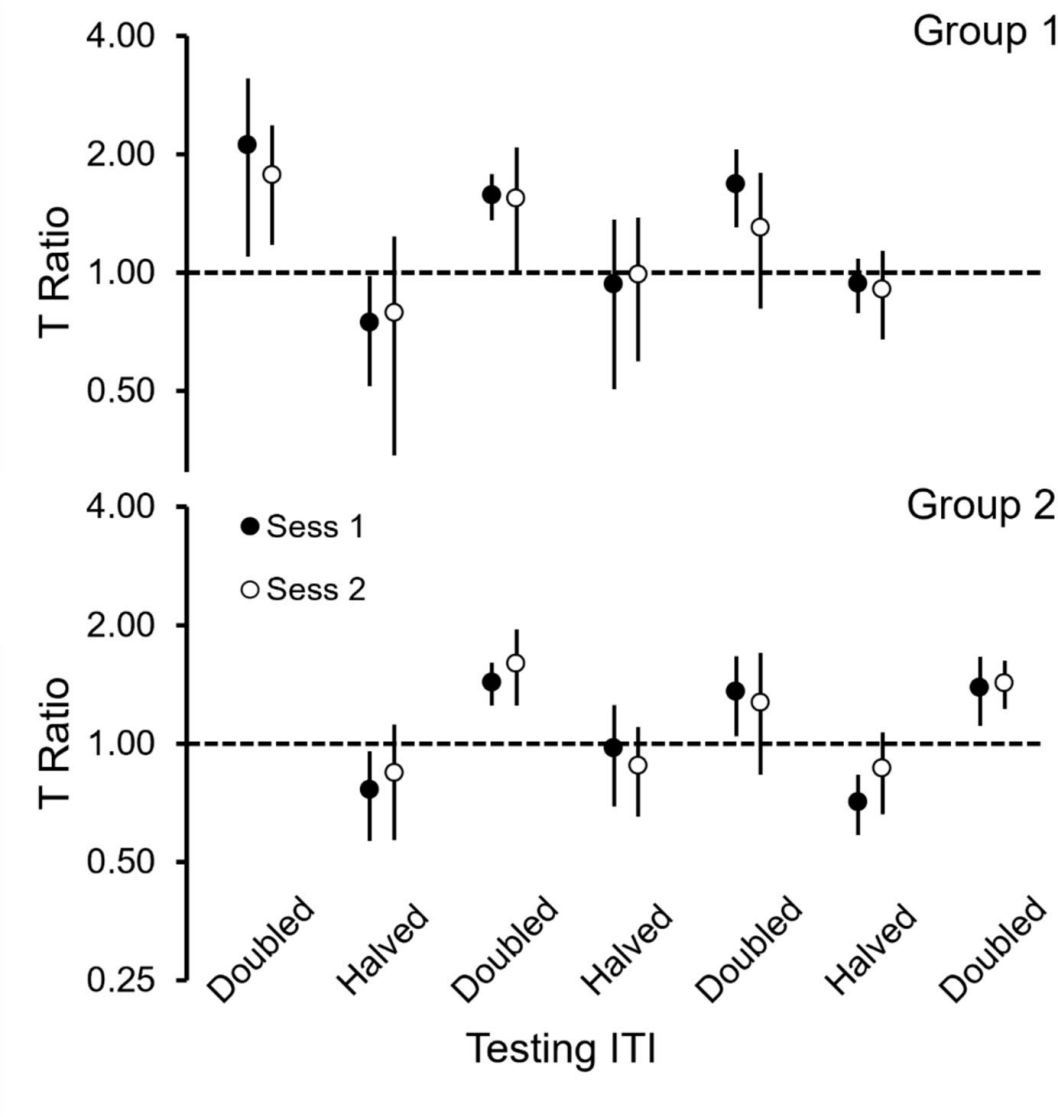
T Ratios across the Experiment for Groups 1 (Top) and 2 (Bottom). *Note.* For each group, the six pairs of filled and empty circles show the average T Ratio (and 95% CI) for the two test sessions that followed each experimental condition, with condition defined by the ITI used in testing (see X-axis). Doubled and Halved ITIs averaged 26 s and 13 s, respectively. Note logarithmic Y axis

Figure 4 shows the average T Ratio for the two groups across the experiment. The ratios remained relatively close to 1 in the halved ITI condition, but not in the doubled ITI condition. Also, the deviations from 1 were systematic, for the ratios were consistently greater than 1 when testing with doubled ITIs and consistently less than 1 when testing with halved ITIs. The T Ratios from the second and third replications deviated from 1 less than the T Ratios from the first replication. The two test sessions yielded similar results.

The foregoing impressions were consistent with the results from the mixed ANOVA, which revealed a main effect of the ITI (*F*(1,12) = 83.17, *p* < 0.001), with a strong effect size (${\eta }_{p}^{2}=$ 0.87), and an interaction effect of Replication and ITI (*F*(2,24) = 6.69, *p* = 0.005) with a moderate effect size (${\eta }_{p}^{2}=$ 0.36). No other effect was significant.

The fact that the T Ratio was greater than 1 when the ITI was doubled, means that the starlings preferred S1 for an interval longer than in training. With trials proceeding at a lower rate, maintaining a preference for S1 for a longer interval suggests the influence of nontemporal, local or numerical cues. In the same vein, the fact that the T Ratio was sometimes less than 1 when the ITI was halved, means that the preference for S1 lasted less in testing than in training. But because the trials occurred at a higher rate, preferring S1 for a shorter interval also suggests local or numerical control (i.e., a starling reversing preference around trial 40 would show a preference for S1 for a period shorter than in training and a T Ratio significantly less than 1).

**Fig. 5:**
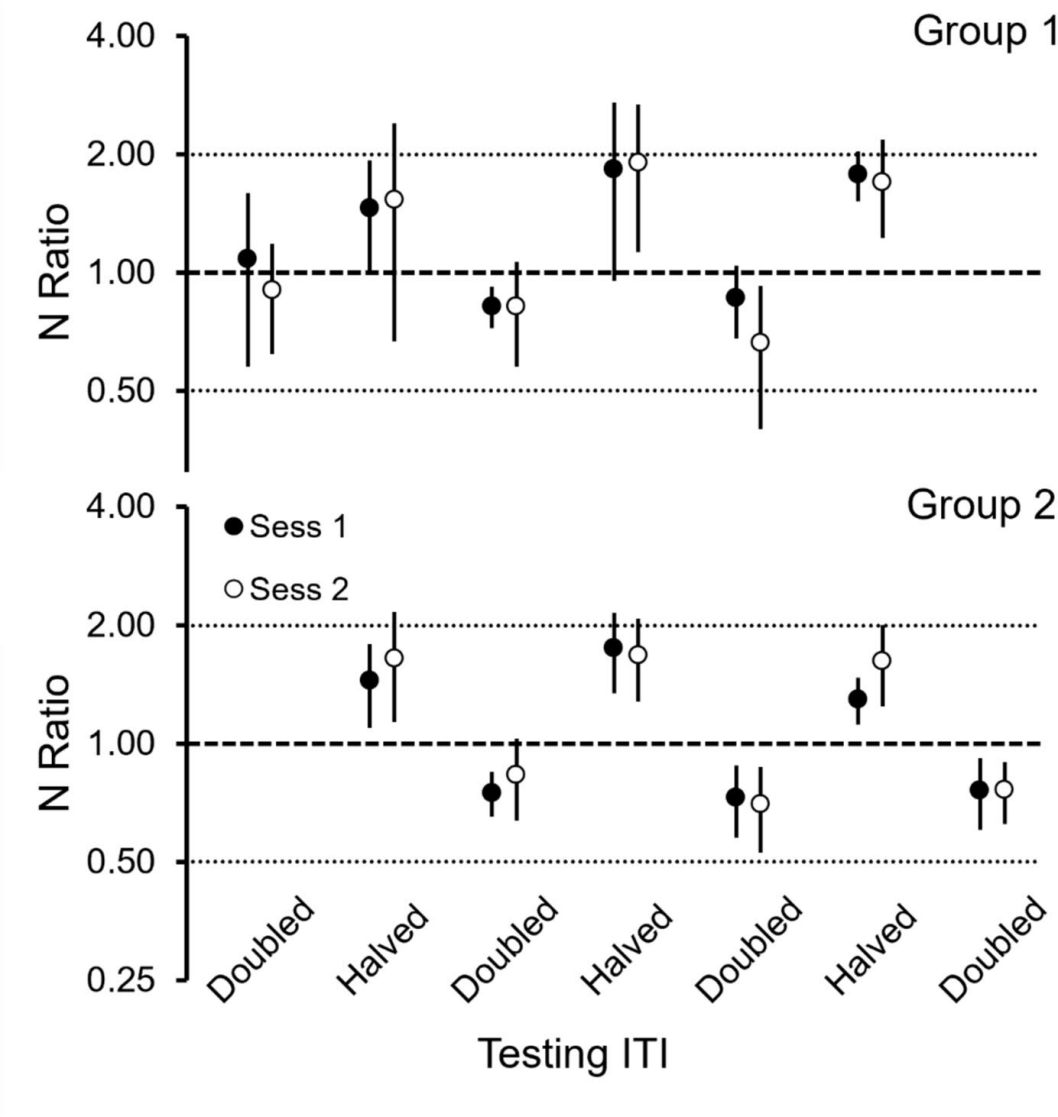
N Ratios across the Experiment for Groups 1 (Top) and 2 (Bottom). *Note.* For each group, the six pairs of filled and empty circles show the average N Ratio (and 95% CI) for the two test sessions that followed each experimental condition, with condition defined by the ITI used in testing (see X-axis). Doubled and Halved ITIs averaged 26 s and 13 s, respectively. Note logarithmic Y axis

Figure 5 shows the average N Ratio across the experiment. The temporal hypothesis predicts N Ratios close to 0.5 and 2.0. For both groups, the ratios tended to be greater than 1 when testing with the halved ITI and less than 1 when testing with the doubled ITI. The only exception was the N Ratio slightly above 1 in the very first test session of Group 1 (leftmost filed circle). The mixed ANOVA of the N Ratios revealed a main effect of the ITI (*F*(1,12) = 82.99, *p* < 0.001) with a strong effect size (${\eta }_{p}^{2}=$ 0.87). No other effect was significant.

It is apparent from Fig. 5 that the deviations of the N Ratios from 1 were not of the same magnitude. When testing with halved ITIs, the N Ratio averaged 1.63, which represents a change of 63% relative to the change predicted by the timing hypothesis (i.e., 100*(1.63–1.)/(2–1))), whereas when testing with doubled ITIs, the N Ratio averaged 0.8. which represents a change of 40% relative to the change predicted by the timing hypothesis (i.e., 100*(0.8–1)/(0.5–1)).

**Fig. 6:**
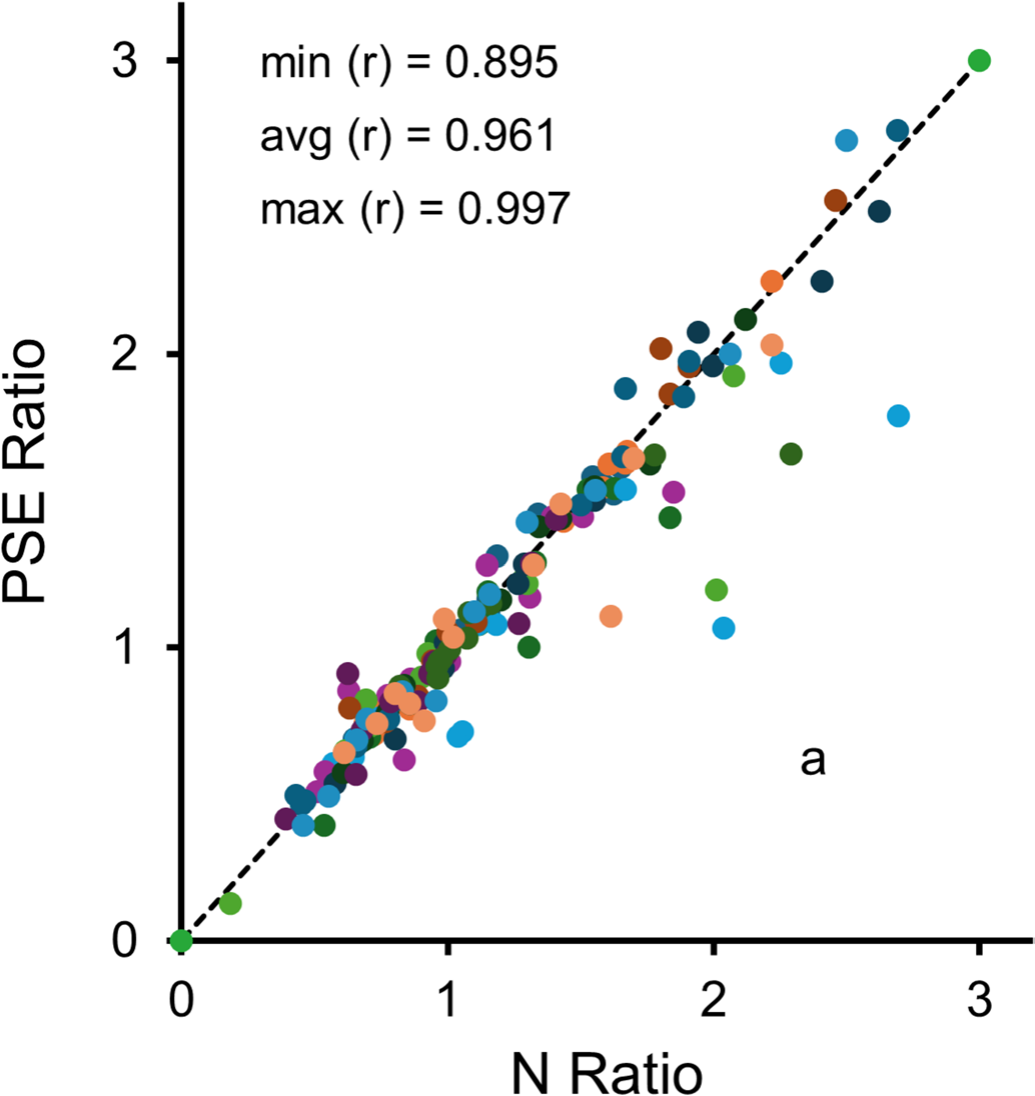
PSE Ratios obtained from the psychometric function plotted against the N Ratios obtained from the trial-based cumulative difference records. *Note.* Each subject contributed 12 points; r stands for the individual correlations. The minimum, average, and maximum r statistics exclude outlier “a”. With the outlier included, the minimum changes to.37 and the average changes to.92

The N Ratios obtained from the cumulative difference records and the PSE Ratios obtained from the psychometric function behaved similarly. With a few exceptions, their correlation was always strong. Figure 6 plots the PSE Ratios against the N Ratios for each subject. The correlations ranged from 0.895 to 0.997, with an average of 0.961.

There were two exceptions. First, two PSE Ratios from birds B2 and B12 could not be computed because the psychometric curves never crossed 0.5—the birds maintained a preference for S1 throughout the session. Second, in one test session with the ITI halved (first Long ITI training condition, test session 2), bird B5 showed an N_max_ on trial 96, suggesting a very late preference reversal, but a PSE on trial 25, suggesting a very early preference reversal. This large discrepancy occurred for the following reason: After the first 20 trials, B5 showed a strong positional bias, which caused the psychometric function to cross 0.5 multiple times. Because the PSE equaled the trial of the first crossing, the PSE was very small. In the cumulative difference record, although N_max_ occurred on trial 96 its value was only 40, which means that the bird alternated between S1 and S2 for most of the session. The net effect was a PSE Ratio close to 0.67 and an N Ratio close to 2.37, a clear outlier in Fig. 6 (see point “a”).

The results concerning the T and N Ratios suggest that the starlings did not behave according to a pure strategy. Although the T Ratio was close to 1, as the temporal hypothesis predicts, there was substantial variability in the first exposure, which suggests that temporal control may have been weaker in the initial stages of the experiment. In addition, the deviations from 1 were systematic, and they also suggest a mixture of temporal and nontemporal strategies. The N Ratios and PSE Ratios corroborate this conclusion. The direction of the changes in these measures accorded with the temporal hypothesis, but their magnitude did not. The upward shifts also tended to be larger than the downward shifts.

**Fig. 7:**
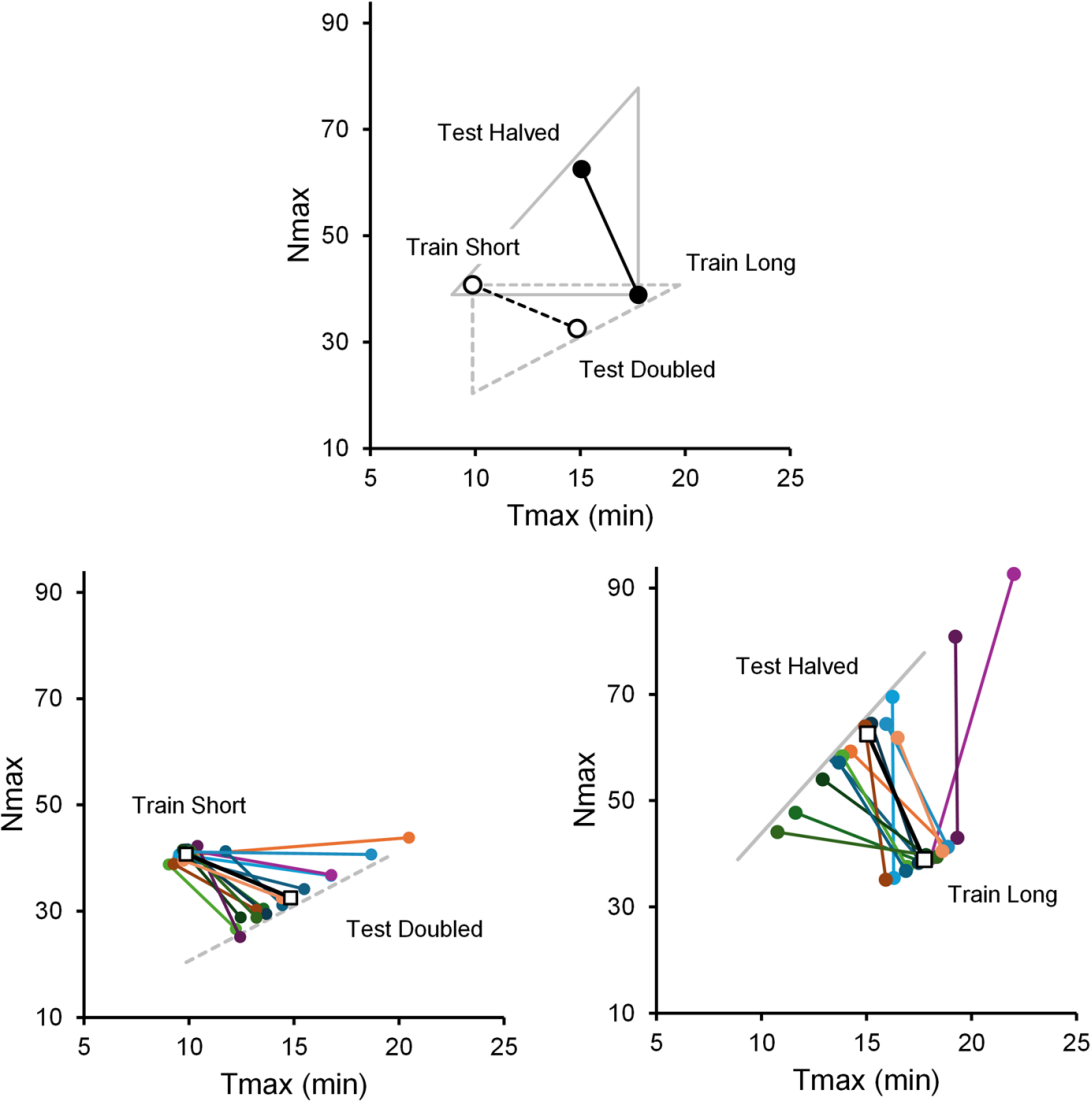
N_max_ as a Function of T_max_ Averaged over All Starlings in the Short ITI Training—ITI Doubled Testing Condition (Open Circles) and Long ITI Training—ITI Halved Testing Condition (Filled Circles). *Note.* The vertical sides of the triangles show (pure) temporal control; the horizontal sides show (pure) local or numerical control. The hypotenuses show a mixture of temporal and nontemporal control. The bottom panels show the individual data. The empty squares replot the average data

The mixture of strategies may be revealed by plotting the average N_max_ against the average T_max_ for the two training conditions, Short and Long. Figure 7 shows the result. Consider a starling exposed to the Long ITI training condition (see point with coordinates T_max_ ≈18 min, N_max_ ≈40). If the starling followed a pure temporal strategy, in the test session (halved ITI) it should preserve T_max_ and double N_max_. Hence, it should move vertically up to point (T_max_ ≈18, N_max_ ≈80). The same starling exposed to the Short ITI training condition (T_max_ ≈10, N_max_ ≈40) should move vertically down when tested with doubled ITI (T_max_ ≈10, N_max_ ≈20). In contrast, a starling following a local or numerical strategy, would change T_max_ and preserve N_max_ when moving from training to testing. Hence, it would move horizontally to the left when testing with halved ITI, and horizontally to the right when testing with doubled ITI. In the experiment, the average movement preserved neither T_max_ nor N_max_. Instead, the average moved in both directions, up and to the left in one case and down and to the right in the other case, suggesting that neither pure temporal control nor pure local or numerical control fit the data.

The bottom panels show the individual data. The variability between and within subjects is substantial. In a few cases, the data points moved almost horizontally or almost vertically, consistent with pure strategies, but in most cases, they moved diagonally, which is more consistent with a mixture of strategies.

### Individual Analysis

**Fig. 8:**
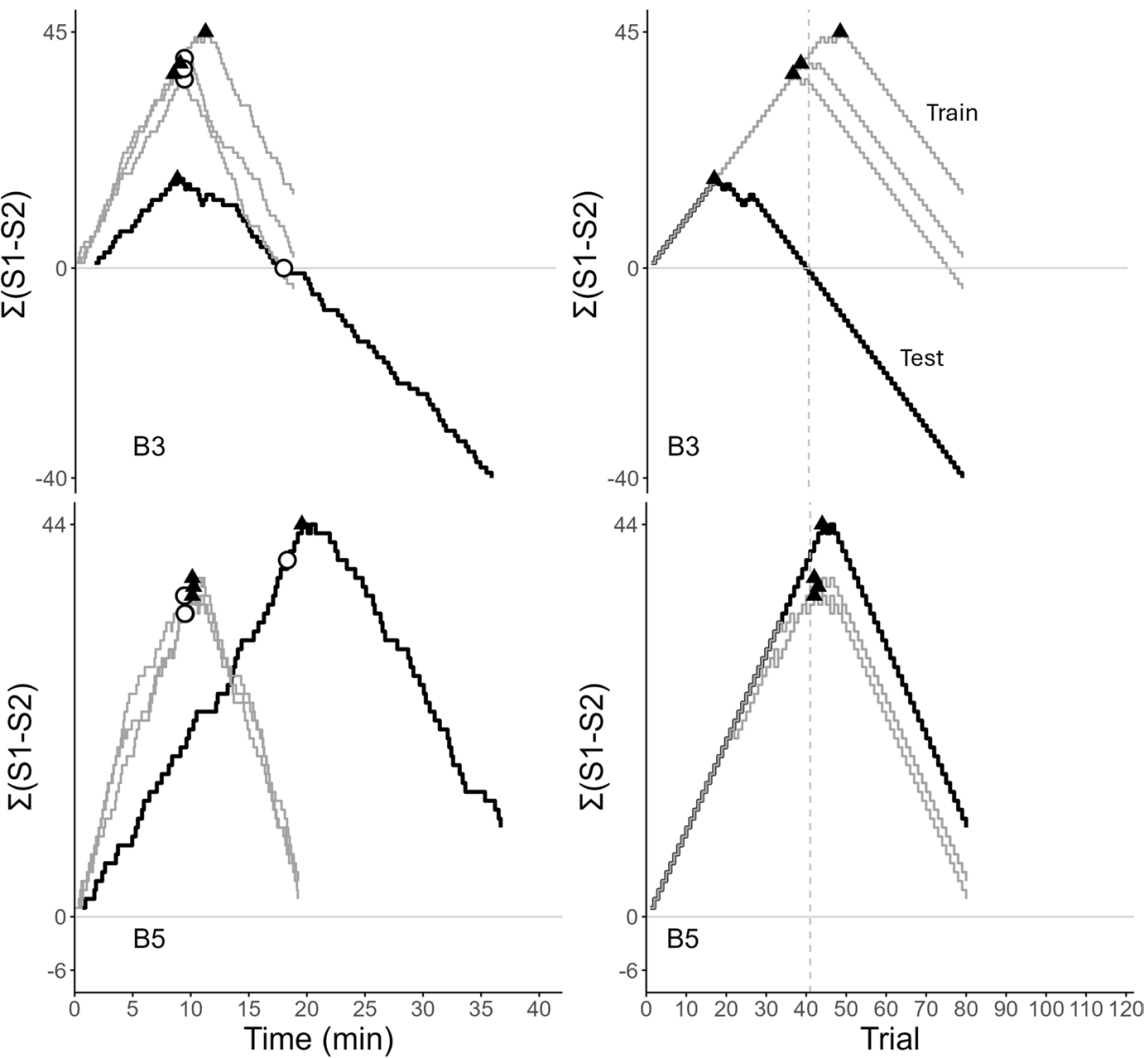
Cumulative Difference Records of Starlings B3 and B5, Plotted against Time (Left) or Trial (Right). The records show three training sessions (grey) before the test session (black). Triangles indicate the peak of preference, and the empty circle and vertical dashed line mark the reversal trial

**Fig. 9:**
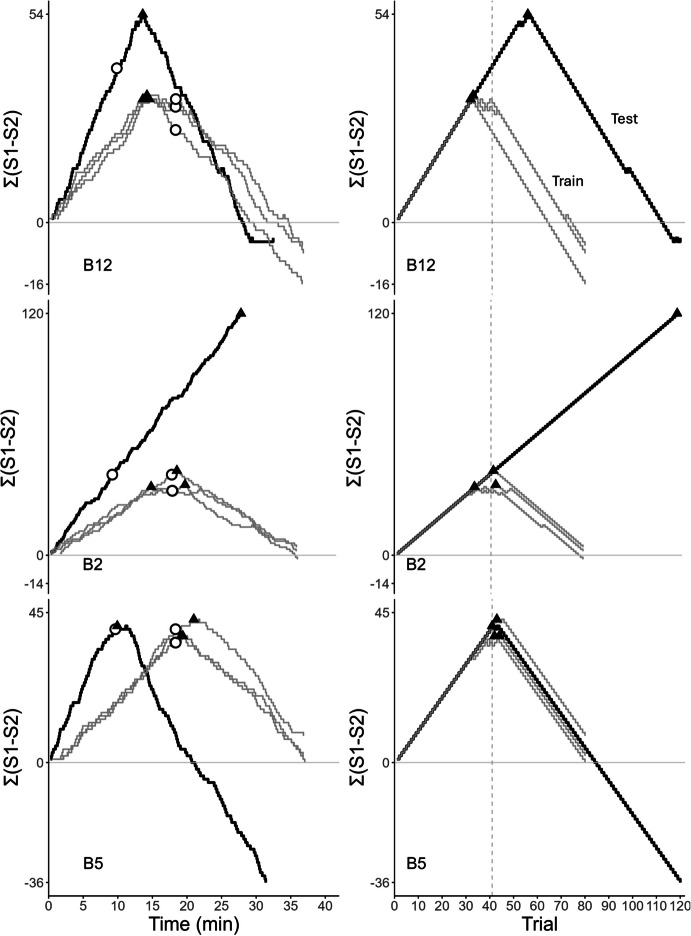
Cumulative Difference Records of Starlings B2, B5, and B12, Plotted against Time (Left) or Trial (Right). *Note.* The records show three training sessions (grey) before the test session (black). Triangles indicate the peak of preference, and the empty circle and vertical dashed line mark the reversal trial

These different strategies were further revealed by the analysis of the cumulative difference curves. Figures 8 and 9 illustrate them. In Fig. 8, the records were produced by starlings B3 and B5 in three Short ITI training conditions and in the ensuing test session. For both birds, the training sessions lasted approximately 20 min, indicating that one trial lasted 15 s on average (ITI ≈13 s + response latencies ≈2). The reversal point occurred around 10 min into the training sessions and the peak of preference, T_max_, occurred close to the reversal moment (cf. triangles and circles). The test session (with the Long ITIs) lasted approximately 37 min for both starlings, but T_max_ varied significantly between them. For B3, T_max_ occurred almost at the same moment it occurred in training (≈10 min) and well before the reversal, a result consistent with the temporal hypothesis. In contrast, for B5, T_max_ occurred shortly after the reversal (≈20 min) and much later than in training, a result inconsistent with the temporal hypothesis. The trial-based records corroborate these conclusions. For B3, N_max_ decreased by a factor of 2 during the test session, as the temporal hypothesis predicts, but for B5, N_max_ hardly changed. B5 reacted to the contingency change on trial 41, reversing its preference after a few nonreinforced choices of S1, which suggests the influence of local, nontemporal cues.

Figure 9 shows different patterns from the Long ITI training condition. Subject B12 shows anticipation errors during the training sessions, with T_max_ ≈14 min. In the test session, T_max_ did not change appreciably but it occurred after the reversal. As the top right panel shows, N_max_ changed from ≈34 in training to 56 in the test session, somewhat consistent with the timing hypothesis. In contrast, B2 presents a different pattern during the test, exclusive choices of S1. Because in the test session choices of S1 are always reinforced, this pattern suggests strong control by nontemporal, local cues. Unlike these two performances, B5 shows, both in the training and the test session, a switch of preference after the reversal. However, during the test session, the reversal is not signaled (both S1 and S2 are always reinforced after trial 40). The switch could have been controlled by a numerical cue. In summary, the analysis of the cumulative difference records revealed different strategies in different starlings as well as different strategies in the same starling when exposed to different conditions.

## Discussion

Previous studies with the MSR task found that starlings, like pigeons, tend to anticipate the reversal trial seemingly using time from the beginning of the session as a cue. To test this temporal hypothesis, researchers have used the following strategy. First, train the subjects with the reversal at a fixed trial (${n}^{*}=41$ in the standard MSR task) and a roughly constant time into the session, ${t}^{*}={\delta }_{Train}\times {n}^{*}$, where ${\delta }_{Train}$ is the ITI during the Training session. Second, change the ITI in a test session such that a significantly different trial occurs at ${t}^{*}$. If the ITI is doubled (i.e., ${\delta }_{Test}=2{\delta }_{Train}$), the trial that will occur at time ${t}^{*}$ will be $n={t}^{*}/{\delta }_{Test}={n}^{*}/2$; if the ITI is halved (i.e., ${\delta }_{Test}={\delta }_{Train}/2$), the trial that will occur at time ${t}^{*}$ will be $n=2{n}^{*}$. If choice is under temporal control, the animal will reverse preference after half as many trials as before, in the former case, and after twice as many trials as before, in the latter case.

The logic is straightforward, but the test becomes problematic if novel, extraneous factors intrude during the test session, causing significant generalization decrement (Capaldi, 1966). Two such factors may be the long series of extinction trials during test session with ${\delta }_{Test}={\delta }_{Train}/2$ (i.e., in which reversal is predicted twice as late), and the distinctly different ITI duration introduced during the test session. Machado et al. (2023) and Soares et al. (2023) controlled the first factor by adjusting the reinforcement contingencies during the test session. In the present study, we attempted to control also the second factor by using variable ITIs in training and in testing, ITIs coming from distributions with different means, but overlapping ranges. The adjusted reinforcement contingencies and the variable ITIs should reduce generalization decrement. With an improved test, we expected preference reversals to occur closer than in previous studies to the 0.5 and 2.0 values predicted by the temporal hypothesis.

The results showed that starlings learned the MSR task, even with variable short or variable long ITIs. They produced orderly psychometric functions with the typical ogival shape, denoting good discrimination at the beginning and end of the training sessions, with anticipation and perseveration errors, and a PSE close to the reversal trial (overall PSE = 39.6(± 3.14). In test sessions, when the ITI was doubled, the psychometric function shifted to the left, and when the ITI was halved, it shifted to the right. Across groups, replications, and test sessions, the direction of the shifts was highly consistent with the temporal hypothesis.

However, the magnitude of the shifts was smaller than predicted. When the test trials proceeded twice as fast as in training, the PSE increased by 50%, whereas in two previous studies with constant ITI’s the PSE had increased by 75% to 100% (Machado et al., 2023, with starling; Santos et al., 2021, with pigeons). When the trials occurred twice as slow as in training, the PSE decreased by 22%, whereas in the previous studies with constant ITIs they had decreased by 29% to 50% (Machado et al., 2023; McMillan & Roberts, 2012; Smith et al., 2017; Soares et al., 2020; Santos et al., 2021). The variable ITIs intended to reduce generalization decrement did not engender larger shifts.

The analysis of the cumulative difference records allowed us to see how preference changed as a function of both time and trials into the session. We obtained two indexes from them, the time and the trial when the preference for S1 reached its peak, T_max_ and N_max_, respectively and then we formed the T Ratio (= T_max_ from Test/T_max_ from Training) and N Ratio (= N_max_ from Test/N_max_ from Training). These ratios shed light on the birds’ potential response strategies in the MSR task. A bird that behaved solely according to the temporal hypothesis would preserve T_max_ when going from training to testing and therefore its T Ratio would remain close to 1. As for N_max_, it would double when test trials proceed twice as fast as in training (N Ratio ≈2), and it would halve when trials proceed twice as slow as in training (N Ratio ≈0.5). But suppose that the bird relied solely on nontemporal, numerical or local cues such as response-outcome pairs or runs of extinction choices (e.g., Rayburn-Reeves et al., 2011; Santos & Sanabria, 2020; Santos et al., 2021; Smith et al., 2016; Zentall, 2020). In this case, T_max_ and N_max_ would switch roles—N_max_ would be the invariant (N Ratio = 1) whereas T_max_ would halve when test trials proceed twice as fast as in training (T Ratio ≈0.5), and it would double when trials proceed twice as slow as in training (T Ratio ≈2).

The data agreed with neither pure strategy. The T Ratio showed systematic deviations from 1 as a function of ITI condition (Fig. 4), which goes against a pure temporal hypothesis. But the N Ratio also showed even greater systematic deviations from 1 (Fig. 5) according to the ITI condition, which goes against a pure local or numerical hypothesis. A combination of strategies seems more consistent with the overall quantitative data (Fig. 7) as well as with the more qualitative analyses of individual cumulative difference records (Fig. 8 and 9).

Perhaps temporal and nontemporal, global and local, exogenous and endogenous combine, compete, or trade-off to regulate choice in the MSR task (McMillan et al., 2017; Rayburn-Reeves et al., 2017; Cook et al., 2021; Machado et al., 2023; Soares et al., 2023). If these sources of control are always active, albeit to various degrees, and affect choice with different weights, the challenge will be to isolate their separate contributions experimentally and to model their joint action theoretically (see Santos et al., 2021). Computational approaches, such as those proposed by Nosofsky et al. (2024) in categorization and memory tasks, may provide a useful framework for modeling these interactions (see also Santos & Sanabria, 2020, and Santos et al., 2021).

Our results also suggest that with multiple replications, temporal control appears to have improved, particularly in Group 1, which exhibited larger shifts across repeated conditions (cf. Figures 2–5 as well as the significant ITI x Replication interaction revealed by the ANOVA). This improvement may be due to reduced novelty with repeated exposure to the same intervals. Some birds’ strategies may have changed over time.

Variable ITIs not only failed to expose stronger temporal control, as the argument based on generalization decrement predicted, but may even have reduced temporal control. Why that may have happened remains unclear—perhaps the steady trial pacing, and hence of eating, engendered by constant ITIs increases the salience of time or of one of its endogenous correlates (e.g., satiety cues). Be that as it may, this finding raises an interesting question. Anticipation errors (as revealed by the first part of the psychometric functions in Fig. 2) are prima facie evidence of temporal control. It would seem to follow that an animal showing them during training sessions would reveal temporal control in the test session (e.g., a shifted PSE). Our data show that this is not necessarily the case; the animal that anticipates in a training session may follow local cues in a test session. Perhaps then the converse argument may carry some weight. A steep psychometric function, one without anticipation errors and only a few perseveration errors, is not necessarily incompatible with temporal control: a rat showing near-optimal performance (Rayburn-Reeves et al., 2013; Santos & Sanabria, 2020; Smith et al., 2016) may nevertheless reveal temporal control (shifted PSE) when trial spacing varies from training to testing. Evidence for stimulus control may require direct manipulation of the putative control cue, as suggested by the results of Rayburn-Reeves et al. (2017) and Cook et al. (2021).

Besides testing the temporal hypothesis with variable ITIs, the present experiment made two other contributions. First, it explored new ways to analyze MSR data, the time- and trial-based cumulative difference records, and the two metrics, the time and trial of the peaks of preference and how these change from training to testing. Second, the experiment contributed to the comparative study of performance in the MSR task (McMillan et al., 2017) by studying starlings, characterizing their response strategies, and comparing them to the strategies of other species.

In summary, although the timing hypothesis accounts for main aspects of the data and clearly identifies one of the cues that guide the behavior of starlings and pigeons in the midsession reversal task, our results show that other, nontemporal, local or perhaps even numerical cues also play a role. Behavioral adaptation in this serial learning task is more complex than initially thought, for it is likely to involve a multiplicity of cues, perhaps hierarchically organized, and flexibly deployed in dynamic environments. Future research should attempt to identify these cues (e.g., time into the session, number of trials, responses and outcomes from previous trials, or satiation levels), how the prior likelihood of their engagement varies across species, how their relative weights change with individual reinforcement histories, and how they combine to determine behavior.

## Data Availability

The data supporting the findings of this study are accessible through the University of Aveiro and William James Center for Research, but their availability is limited, meaning they are not publicly accessible. However, the data can be obtained from the authors upon reasonable request.
